# Comparative effectiveness of first-line antihypertensive drug classes on the maintenance of estimated glomerular filtration rate (eGFR) in real world primary care

**DOI:** 10.1038/s41598-023-48427-4

**Published:** 2023-12-01

**Authors:** Qiao Gao, Ngiap Chuan Tan, Mong Li Lee, Wynne Hsu, Jason Choo

**Affiliations:** 1 Institute of Data Science, National University of Singapore, Singapore, Singapore; 2 SingHealth Polyclinics, Singapore, Singapore; 3 School of Computing, National University of Singapore, Singapore, Singapore; 4 Department of Renal Medicine, Singapore General Hospital, Singapore, Singapore

**Keywords:** Hypertension, Chronic kidney disease

## Abstract

Renin-angiotensin system inhibitors (RASi), particularly angiotensin-converting enzyme inhibitors (ACEIs) and angiotensin II receptor blockers (ARBs), are commonly used in the treatment of hypertension and are recommended for kidney protection. Uncertainty remains about the effectiveness of RASi being used as first-line antihypertensive therapy on eGFR maintenance compared to its alternatives, especially for those with no or early-stage chronic kidney disease (CKD). We conducted a retrospective cohort study of 19,499 individuals (mean age 64.1, 43.5% males) from primary care in Singapore with 4.5 median follow-up years. The study cohort included newly diagnosed individuals with hypertension (whose eGFR was mainly in CKD stages G1-G2) and initiated on ACEIs, ARBs, beta-blockers (BBs), calcium channel blockers (CCBs) or diuretics (Ds) as first-line antihypertensive monotherapy. We compared the estimated glomerular filtration rate (eGFR) curve before/after the drug initiation over time of patients under different drug classes and analyzed the time to declining to a more advanced stage CKD. Inverse probability of treatment weighting (IPTW) was used to adjust for baseline confounding factors. Two key findings were observed. First, after initiating antihypertensive drugs, the eGFR almost maintained the same as the baseline in the first follow-up year, compared with dropping 3 mL/min/1.73 m^2^ per year before drug initiation. Second, ARBs were observed to be slightly inferior to ACEIs (HR = 1.14, 95% CI = (1.04, 1.23)) and other antihypertensive agents (HR = 1.10, 95% CI = (1.01, 1.20)) in delaying eGFR decline to a more advanced CKD stage in the study population. Our results showed that initiating antihypertensive agents can significantly maintain eGFR for those newly diagnosed patients with hypertension. However, RASi may not be superior to other antihypertensive agents in maintaining eGFR levels for non-CKD or early stages CKD patients.

## Introduction

Hypertension affects around 1.2 billion people worldwide^1^ and is strongly associated with the prevalence of CKD^2^. In patients with newly diagnosed hypertension, five antihypertensive drug classes [angiotensin-converting enzyme inhibitors (ACEIs), angiotensin II receptor blockers (ARBs), beta-blockers (BBs), calcium channel blockers (CCBs) and diuretics (Ds)] are widely recommended by clinical guidelines as first-line blood pressure lowering drugs, either in combination or as monotherapy^3–8^. Among them, ACEIs and ARBs are recommended by major clinical practice guidelines for their kidney protection benefits^4,9^, aside from lowering blood pressure and cardiovascular disease protection. These drugs are preferentially prescribed to those with CKD and type-2 diabetes mellitus.

The evidence supporting the superiority of ACEIs and ARBs over other drug classes originated mainly from clinical trials and utilized end-stage kidney disease (ESKD) and doubling of baseline serum creatinine concentration as clinical outcomes^10–12^. Due to the long progression time of chronic kidney disease, ESKD is more likely to be observed in patients already with later stages chronic kidney disease, i.e., eGFR ≤ 45 mL/min/1.73 m^2^. For those newly diagnosed with hypertension and with eGFR in an early stage of CKD (eGFR > 60 mL/min/1.73 m^2^), uncertainty remains on whether ACEIs and/or ARBs are superior to other antihypertensive drug classes on kidney protection. Another pertinent question relates to whether ACEIs or ARBs confer better kidney function when they are initiated as monotherapy in patients with hypertension and vascular diseases. The majority of these patients are asymptomatic and are treated in primary care.

In order to determine the effectiveness of ACEIs and ARBs on kidney function, we leverage on a real-world primary care clinical dataset to conduct a comparative analysis of five first-line antihypertensive drug classes as monotherapies on the maintenance of eGFR over time. The study outcomes analyzed included eGFR value change over time after drug initiation and time to eGFR decline to a more advanced CKD stage.

## Methods

### Study setting and data source

This retrospective cohort study was conducted using electronic medical records (EMR) from SingHealth Polyclinic (SHP), a cluster of public primary care clinics (polyclinics) in the eastern region of Singapore. This cosmopolitan island state at the centre of Southeast Asia had a prevalence of hypertension of 21.5% in 2017 of its adult residents living in a densely populated, urban environment treated by a mixture of private and public primary care clinics^13^.

SHP includes eight separately located polyclinics, which managed over 2.3 million multi-ethnic Asian patient attendances in 2021. Each polyclinic serves about 450 to 1100 patients across all ages during office hours for each work day, with their clinical care documented in EMR.

All adult patients with a clinical diagnosis of hypertension were identified from the SHP EMR from January 1, 2010 to December 31, 2020. Eligible patients' clinical data, including demographics, laboratory test results, and prescribed medications, were extracted, audited, and de-identified by an approved trusted third party before analysis. All methods were performed in accordance with relevant guidelines and regulations.

### Cohort design and data processing

Adult patients with hypertension initiated on a first-line antihypertensive drug as monotherapy from these five drug classes, i.e., ACEIs, ARBs, BBs, CCBs and Ds were identified and extracted from the entire data set. The drugs considered in each class are listed in Table S1.

The study cohort constructed followed a retrospective, comparative new-user design^14–16^. More specifically, we first identified when the individuals were first exposed to an anti-hypertensive drug in the selected classes as monotherapy. Then, we required patients to have at least 1 year of previous EHR observation before first exposure to guarantee the treatment initiation. Last, we required a recorded hypertension diagnosis at or within the date preceding treatment initiation. The diagnosis of hypertension was identified based on International Classification of Diseases 10th Revision (ICD-10) codes. In addition, we only considered adult patients (aged 21 years or older), and the patients should have one baseline eGFR, one control eGFR and at least one follow-up eGFR laboratory test. Patients were divided into three groups according to the drug class: those taking ACEIs, those taking ARBs, and those taking either BBs, CCBs or Ds. Those taking BBs, CCBs and Ds were grouped together as "OTHERS" based on evidence from previous literature showing that they were less effective compared to RASi in kidney protection^17^. Patients who were prescribed concurrently with Sodium-GLucose coTransporter-2 (SGLT2) inhibitors such as empagliflozin and dapagliflozin in the EMR were excluded because of their known kidney protective effects^18–20^.

The observation period began from the date of drug initiation (index date) to the end date of the same drug prescribed in the EMR. More specifically, the observation would end with any one of the following occurrences: (1) switch to another antihypertensive drug, or (2) addition of another antihypertensive drug, or (3) no refill prescription of the current antihypertensive drug within 1 year from the last prescription.

The eGFR value was computed from serum creatinine values reported from laboratory reports in the EMR using the CKD-EPI Creatinine Equation 2021^21^. Serum creatinine was analyzed using the kinetic Jaffe method on the Cobas c702 analyzer (Roche, Mannheim, Germany). The creatinine assay calibration is traceable to the isotope dilution mass spectrometry (IDMS) reference creatinine method.

**Figure 1:**
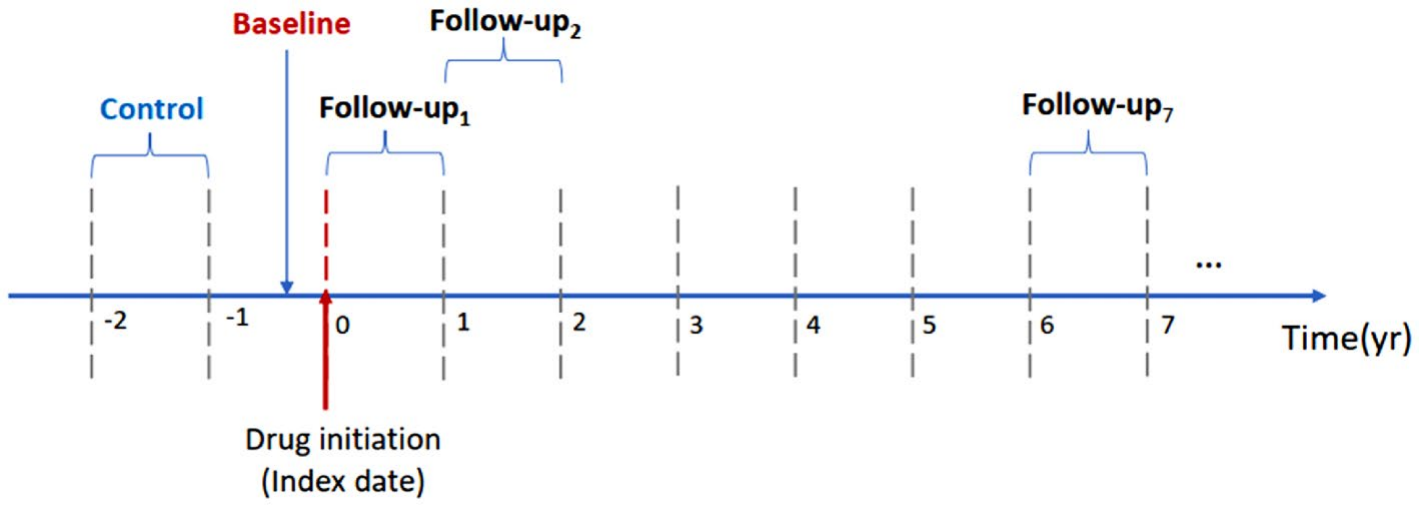
Timeline to extract the baseline, follow-up and control eGFR values.

The baseline eGFR of each patient was defined as the last eGFR measured within the 1-year period before and including the index date. The control eGFR is defined as the eGFR measured within the 1-year to 2-year period before the index date. The follow-up eGFR sequences are those measured consecutively after the index date to the end of the observation period. In the polyclinics, the eGFR of the patients are tied to a laboratory panel test (which includes kidney function) performed once a year, a single eGFR reading was extracted every year after the index date. In the situation where multiple eGFRs were measured in a single year, we computed the mean eGFR (for control and follow-ups) or the last eGFR (for baseline) as the indicator of kidney function for that year. For lapses in eGFR assessment for any patient, we used linear interpolation to estimate the eGFR value (Fig. 1).

### Statistical analysis and outcomes

The effectiveness evaluation was conducted pairwise among ACEIs, ARBs and OTHERS antihypertensive drug classes. Inverse probability treatment weighting (IPTW) was used to control the confounding factors in baseline characteristics. The propensity score was estimated using multivariate logistic regression with l2-regularization. Stabilized weights were used to increase precision by adding the marginal probability to the numerator of the weights. Weights were considered appropriate if the standardized mean difference (SMD) between treatment groups was < 0.1 (Tables S3–S5).

After appropriate weightage was assigned, the following outcomes were estimated. First, the mean and 95% confidence interval of eGFR differences from control to baseline, and also from eGFR at first follow-up value to baseline was computed. This was aimed at evaluating the effectiveness of drug initiation.

Next, patients prescribed the same drug for at least 5 years were selected in each group, and the eGFR differences to baseline over time were compared. In addition, we also estimated the eGFR curve under the case when patients did not initiate the drug at index date by assuming the eGFR would decline with the same slope as that between control and baseline values over time.

Finally, the association between eGFR progression and drug classes was evaluated during the observation period. The eGFR progression was defined as the time from drug initiation to when follow-up eGFR values declined to a more advanced stage (e.g. stage G1 to stage G2 or stage G1 to stage G3) compared to baseline at two consecutive visits. The association was estimated by multivariate Cox proportional hazard regression. The study population was grouped according to their demographic characteristics and clinical status at baseline, including gender, age, systolic blood pressure, eGFR stages, diabetes mellitus, kidney disease, and macrovascular disease. The regression study was repeated in the subgroups of the entire population.

The baseline variables considered in both propensity score estimation and Cox proportional hazard regression were age, gender, ethnic group, laboratory measurements [including HbA1c, low density lipoprotein-cholesterol (LDL-C), systolic and diastolic blood pressure and the body mass index (BMI), and eGFR stages], comorbidity status, complication status, and medication prescription. All analyses were performed using Python 3.8.12.

### Ethics approval and consent to participate

Ethics approval was obtained from SingHealth Centralized Institution Review Board (CIRB) in 2019 (SingHealth CIRB Reference: 2019/2604). Requirement of written consent was waived by the SingHealth CIRB as it was deemed impracticable while privacy risks were mitigated through the use of de-identified data.

## Results

### Study cohort and baseline characteristics

From 124,753 candidate patients newly diagnosed with hypertension, we identified 19,499 patients with mean age 64.1 who satisfied the selection criteria and were included in the study population (Fig. S1), of which 43.5% were male. Among them, 3832(19.6%) patients were initiated with ACEIs, 3171(16.3%) with ARBs and 12,496 (64.1%) with OTHERS drug classes (BBs, CCBs, Ds) as first-line monotherapy treatment for hypertension. The median duration of follow-up was 4.5 (IQR = [2.1, 8.0]) years. The median frequency of eGFR measurement was 1.09 times per year (IQR = [0.96, 1.45]).

The baseline characteristics for patients under different first-line antihypertensive treatments are given in Table 1. Across all three cohorts, patients were similar in the age distribution, baseline laboratory values, diagnosis of hyperlipidaemia and anti-hyperlipidaemic medications. The gender distribution was different between each drug class pair. The majority (> 80%) of patients in each group were in CKD stages G1 and G2, with very few patients (< 1%) in CKD stages G4 and G5. Comparing those initiated with ACEIs or ARBs with those initiated on OTHERS, patients with ACEIs or ARBs were more likely to have diabetes, nephropathy and retinopathy. Thus, more patients with ACEIs or ARBs were on anti-diabetic medications at baseline. The patient profiles of those under ACEIs and ARBs were very similar, except for the cohort with ACEIs, which had slightly more male and diabetic patients.

**Table 1:** Baseline characteristics of patients in study cohort.

Characteristics	ACEIs	ARBs	OTHERS
N	3832	3171	12,496
Age (year)	63.4 ± 10.5	63.4 ± 10.6	64.5 ± 10.3
**Gender**			
Male	2096 (54.7)	1484 (46.8)	4910 (39.3)
Female	1736 (45.3)	1687 (53.2)	7586 (60.7)
**Race**			
Chinese	2940 (76.7)	2504 (79.0)	10,621 (85.0)
Malay	440 (11.5)	306 (9.6)	1009 (8.1)
Indian	316 (8.2)	226 (7.1)	529 (4.2)
Others	136 (3.5)	135 (4.3)	337 (3.0)
**Baseline lab values**			
HbA1c (%)	7.2 ± 1.0	7.2 ± 0.9	7.1 ± 0.5
LDL-C (mmol/L)	2.6 ± 0.6	2.6 ± 0.6	2.8 ± 0.6
Systolic BP (mmHg)	128.9 ± 15.6	131.1 ± 16.4	132.4 ± 15.3
Diastolic BP (mmHg)	70.7 ± 9.7	71.5 ± 10.1	71.9 ± 9.9
BMI (kg/m^2^)	25.2 ± 4.0	25.7 ± 4.1	25.2 ± 4.0
**Comorbidities**			
Dyslipidemia	3559 (92.9)	2865 (90.4)	10,486 (83.9)
Diabetes	2411 (62.9)	1808 (57.0)	1781 (14.3)
**Complications**			
Macrovascular disease^a^	837 (21.8)	601 (19.0)	2148 (17.2)
Nephropathy^b^	827 (21.6)	666 (21.0)	1371 (11.0)
Retinopathy^b^	694 (18.1)	572 (18.0)	637 (5.1)
Foot complications^b^	100 (2.6)	67 (2.1)	110 (0.9)
**Anti-diabetic medications**			
Biguanides	2198 (57.4)	1609 (50.7)	1594 (12.8)
Sulfonylureas	1295 (33.8)	900 (28.4)	814 (6.5)
DPP4 inhibitors	52 (1.4)	97 (3.1)	44 (0.4)
Alpha glucosidase inhibitors	269 (7.0)	153 (4.8)	125 (0.1)
Insulin	259 (6.8)	182 (5.7)	95 (0.1)
**Anti-hyperlipidemic medications**			
HMG-CoA reductase inhibitors	3451 (90.1)	2779 (87.6)	10,039 (80.3)
Fibric acid derivatives	312 (8.1)	255 (8.0)	778 (6.2)
Cholesterol absorption inhibitors	15 (0.4)	6 (0.2)	19 (0.2)
Bile acid sequestrants	8 (0.2)	3 (0.1)	13 (0.1)
**Baseline CKD stage**			
Stage G1	1805 (47.1)	1622 (51.2)	6200 (49.6)
Stage G2	1584 (41.3)	1194 (37.7)	5543 (44.4)
Stage G3	418 (11.0)	323 (10.2)	726 (5.8)
Stage G4	23 (0.6)	28 (0.9)	24(0.2)
Stage G5	2 (0.0)	4 (0.1)	3 (0.0)

For categorical variables, the values given are n(%). For numerical variables, the values given are mean ± std.

^a^Macrovascular disease refers to coronary and cerebral and peripheral vascular diseases.

^b^Nephropathy, retinopathy and foot complications belong to microvascular disease.

The pairwise comparison of the baseline characteristics of different study cohorts are given in Tables S3–S5. Among them, some attributes, such as gender and diagnosis of diabetes, were highly unbalanced. Using IPTW to remove the confounding factors, all the SMD values after weighting among different treatment groups are smaller than 0.05.

### Drug initiation

For each comparison pair, the differences between the control and the first-year follow-up from the baseline are reported in Table 2. We could see that before initiating the drugs, the eGFR of patients generally declined by about 3 mL/min/1.73 m^2^ per year, whereas after drug initiation, the eGFR value was almost the same compared to the baseline value in the first follow-up year. The differences between the base and comparator in each pair were not significant. Such results demonstrate the kidney protective effect of antihypertensive drugs on delaying eGFR decline, regardless of class.

**Table 2:** Control and follow-up values of drug initiation compared to baseline eGFR value.

Comparison pair		Base		Comparator	
Base	Comparator	$\Delta$ Control	$\Delta$ Follow-up_1_	$\Delta$ Control	$\Delta$ Follow-up_1_
ARBs	ACEIs	3.31 (2.85, 3.37)	− 0.02 (− 0.25, 0.21)	3.11 (2.85, 3.37)	− 0.02 (− 0.25, 0.21)
Others	ACEIs	3.03 (2.91, 3.15)	0.38 (0.27, 0.49)	3.09 (2.69, 3.49)	0.14 (− 0.23, 0.51)
Others	ARBs	2.93 (2.81, 3.04)	0.37 (0.26, 0.47)	2.92 (2.45, 3.38)	− 0.42 (− 0.84, 0.01)

The baseline eGFR was measured within 1 year before the index date, and the follow-up_1_ eGFR of each patient are the value measured in the first follow-up year. $\Delta$ Control and $\Delta$ Follow-up_1_ are the difference of control (or the first-year follow-up) from the baseline. The values given in each cell are the mean and 95% confidence interval.

### eGFR change over time

**Figure 2:**
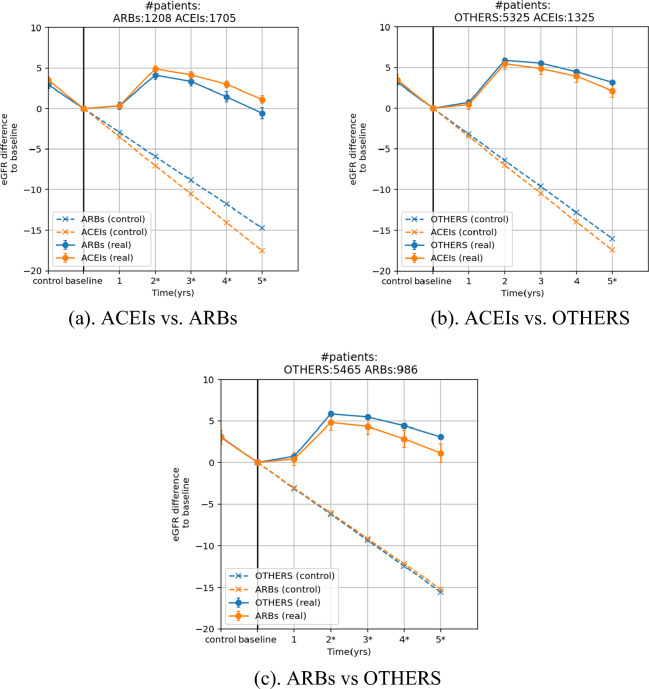
Pairwise comparison of eGFR curve over 5 years. The asterisks besides the time label indicate that the corresponding two means of the real eGFR curve are statistically significant different (P < 0.05). The error bar of each data point depicts the 95% confidence interval. Note that this analysis is conducted in a subset of the whole study population, only including those taking the drugs for more than 5 years. The SMD after weighting in all comparative pairs are smaller than 0.1 (Fig. S3).

Figure 2 shows the eGFR changes for each drug class pair over 5 years. The general trend of different drug class pairs were similar. After drug initiation, the eGFR value maintained in the first year, increased around 5 mL/min/1.73 m^2^ in the second year, and slowly declined until the fifth year. At the end of 5 years, the mean eGFR values were still higher than or near the baseline values, with the differences between drug classes less than 2 mL/min/1.73 m^2^: 1.67 mL/min/1.73 m^2^ (ACEIs vs ARBs, P < 0.05), − 1.01 mL/min/1.73 m^2^ (ACEIs vs OTHERS, P < 0.05), and − 1.96 mL/min/1.73 m^2^ (ARBs vs OTHERS, P < 0.05), respectively.

Compared with the extrapolated control line that resulted in a 15 mL/min/1.73 m^2^ eGFR reduction over 5 years, consistent intake of the antihypertensive drugs showed significant kidney protection in terms of maintenance of eGFR.

### Time to eGFR decline to a more advanced stage

For groups, ACEIs, ARBs and OTHERS, 428 (11.2%), 429 (13.5%) and 941 (7.5%) patients had declining eGFR to a more advanced CKD stage compared with baseline, respectively. The detailed patient numbers of each CKD stage transition group is given in Table S6. In addition, less than 3% of patients in each group had eGFR decline by more than 30% from baseline during the study period (Table S2).

The hazard ratios show no difference between ACEIs and OTHERS in delaying the eGFR to a more advanced CKD stage (HR = 1.03, 95% CI = (0.95,1.12)). However, the ARBs are observed to be slightly inferior to both ACEIs (HR = 1.14, 95% CI = (1.04, 1.23)) and OTHERS (HR = 1.10, 95% CI = (1.01,1.20)) in preventing eGFR decline to a more advanced CKD stage.

**Figure 3:**
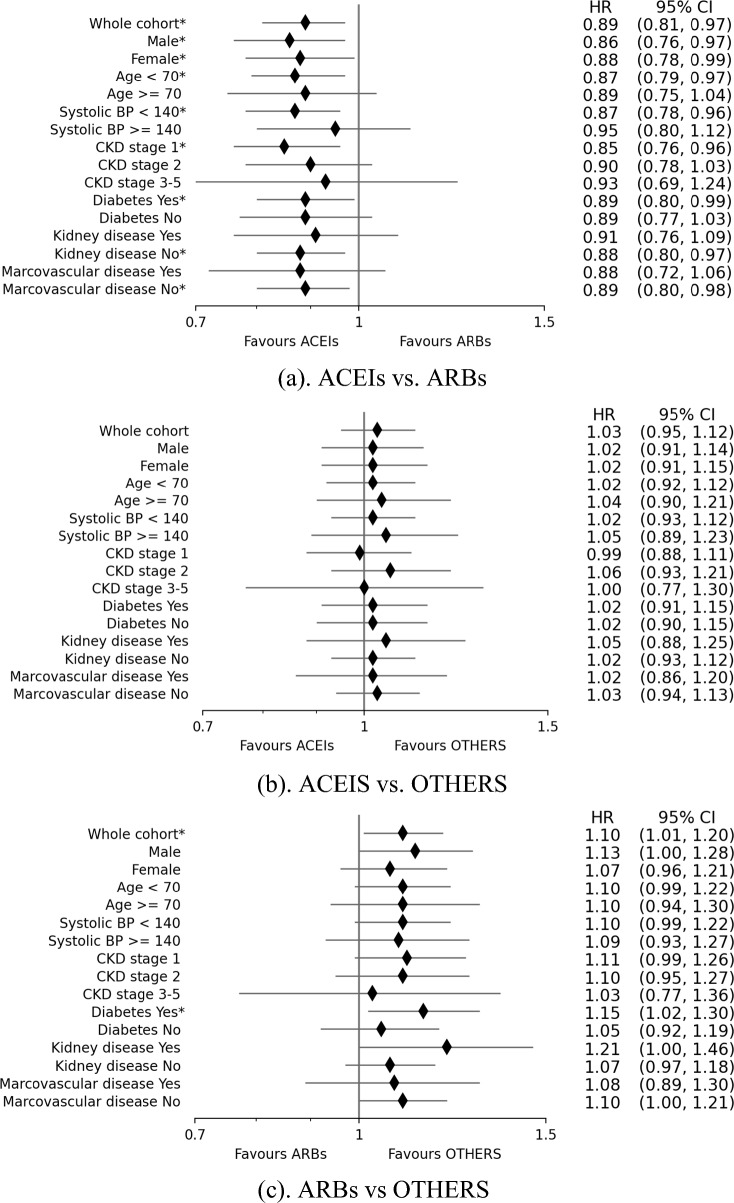
Hazard ratios for eGFR declining to next CKD stage for whole cohort and subgroups divided according to sex, age, systolic blood pressure, CKD stage, diabetes, kidney disease and macrovascular disease. The asterisks besides the labels indicate that the corresponding HR are statistically significant different (P < 0.05).

In subgroup analysis (Fig. 3), ACEIs were observed to be superior to ARBs in patients with lower risks of vascular complications, such as those aged less than 70 years, systolic BP less than 140 mmHg, in CKD stage G1, without kidney disease or without macrovascular disease. Notably, among diabetic patients, ACEIs were superior to ARBs in delaying the eGFR decline to the next CKD stage. By comparing ARBs vs OTHERS, the only statistically significant subgroup was patients with diabetes (HR = 1.15, 95% (1.02,1.30)). Between ACEIs and OTHERS, no statistically significant differences were observed in the subgroups.

## Discussion

### Background and key findings

Leveraging on real-world primary care data, we compared the effectiveness of five common first-line antihypertensive drug classes in maintaining the eGFR of patients who were mostly (> 80%) in CKD stages G1-G2. To the best of our knowledge, such comparison with eGFR value change as an outcome had not yet been investigated. Two main findings were observed in our study. First, for patients with hypertension, initiating an antihypertensive drug in class ACEIs, ARBs, and OTHERS (i.e., BBs, CCBs or Ds) significantly delayed the eGFR decline over time. Second, the ARBs were observed to be slightly inferior to ACEIs and OTHERS in delaying eGFR decline to a more advanced CKD stage, especially for diabetic patients.

### Choice of first-line antihypertensive drugs

Among all patients in this study population, ACEIs and ARBs were more likely prescribed to patients with diabetes or nephropathy (Table 1), which is consistent with current clinical guidelines^3–8^.

### eGFR changing over time

The natural eGFR decline rate of those with hypertension in our dataset is 3 mL/min/1.73 m^2^ per year (Table 2), which is significantly higher than 1 mL/min/1.73 m^2^ per year for the normal population^22^. The rate of eGFR decline in hypertensive individuals varies across studies due to differing study contexts. Polonia et al. found in a retrospective observational study that the average yearly eGFR decline was 3.3 ± 8.2 mL/min/1.73 m^2^ for hypertensive individuals with type II diabetes, and 2.4 ± 7.7 mL/min/1.73 m^2^ for those without type II diabetes^23^. Collard et al., in a post hoc analysis of clinical trial data, reported yearly eGFR decline rates of 0.95–1.23 mL/min/1.73 m^2^ for individuals under standard blood pressure targets and 1.14–1.24 mL/min/1.73 m^2^ for those under intensive targets^24^. Yu et al., in a prospective cohort study, reported yearly eGFR declines of 1.47 to 1.71 mL/min/1.73 m^2^ in hypertensive individuals^25^. Comparatively, our eGFR decline rate aligns closely with the observational study by Polonia et al.^23^ but is more rapid than rates from clinical trials or the prospective study. Potential reasons for this discrepancy could be irregular eGFR measurements in real-world settings, leading to inexact yearly intervals for estimating decline rates, and our estimation was during a phase when patients did not initiate anti-hypertensive drugs, which may accelerate eGFR decline.

In the fifth-year of follow-up, the mean eGFR value of each drug class was still close to baseline eGFR, demonstrating these drugs' effectiveness on eGFR maintenance. Among them, the OTHERS drug class is observed to have the best eGFR maintenance effect, with a higher mean eGFR 1.01 mL/min/1.73 m^2^ (P < 0.05) than ACEIs and 1.96 mL/min/1.73 m^2^ (P < 0.05) than ARBs. Such results are consistent with that in the ALLHAT trial^26^ which showed that CCB had the highest eGFR maintenance at the fourth follow-up year compared to ACEIs and D. The acute eGFR decline after drug initiation^27^ was not observed for ACEIs and ARBs; it may be due to the 1-year interval between laboratory testings, while the acute eGFR decline happened in the first 3 months after the drug initiation^28^.

### eGFR increased in all groups in the second year of follow-up

The cause of this is unclear and was not due to a change in BMI (Fig. S2). Improvement in eGFR was seen in various other studies at varying frequencies. In the MDRD study, GFR improved in 11% of patients^29^ and in a hospital-based nephrology cohort (NephroTest Study Group), measured GFR improved in 15.3% of patients (these patients were less likely to have diabetic glomerulopathy or polycystic kidney disease)^30^. In a separate nephrology clinic referral cohort, significant improvement in eGFR slope was seen in 48.2% of CKD stage G2 patients^31^. A rise in eGFR may mean hyperfiltration in a patient with diabetes, or it may signify the potential occurrence of kidney remodelling or regeneration with regression of kidney fibrosis. Remuzzi et al. in an experimental rat model with spontaneous kidney disease, was able to demonstrate glomerular repair using ACEIs^32^.

### eGFR declined to an advanced stage

Our results showed that ARBs were marginally inferior to ACEIs and OTHERS on delaying eGFR to a more advanced CKD stage. Even though the literature on the effect of antihypertensive drugs on eGFR is limited, some of the previous literature has questioned the additional kidney protective effect of RASi compared to OTHERS with ESKD as an outcome. In a meta-analysis of 127 clinical trials of patients with or without diabetes two decades ago^10^, Casas et al. reported that RASi has a smaller benefit on ESKD than OTHERS with a relative risk of 0.87, CI (0.75, 0.99), but RASi had no additional effect on the eGFR compared to OTHERS during the follow-up period. Such result differences might come from the different drug effectiveness on patients in different CKD stages, considering the patients with no or early-stage CKD have a more challenging time developing ESKD during the follow-up period (Table S2). Moreover, Bangalore et al., in their systematic review and meta-analysis^33^ on patients with diabetes, have shown that RASi resulted in no significant difference in ESKD compared to other antihypertensive agents (relative risk 0.99, 95% CI (0.78, 1.28)), which is consistent with our observation between ACEIs and OTHERS. In studies comparing antihypertensive drug effectiveness on those with CKD (G3-G5), the ACEIs had shown a superior effectiveness than ARBs in preventing ESKD or doubling of serum creatinine level^34,35^, which is also consistent with our observation. This study's novelty comes from utilising the eGFR as an outcome and observing that ARBs were marginally inferior to OTHERS in delaying eGFR decline in those with no or early-stage CKD, which have not been reported yet.

### Contributions

This study contributes to the knowledge that any antihypertensive drug class can potentially be used for kidney protection in newly diagnosed hypertensive patients with no or early-stage CKD in the primary care setting. First, the results affirm that initiating an antihypertensive drug as monotherapy can reduce eGFR decline and maintain the eGFR over time. Second, the results showed that the RASi were not superior to other antihypertensive drugs in eGFR maintenance of patients with no CKD or early-stage CKD, which may suggest that clinicians can select antihypertensive drugs mainly based on the blood pressure lowering effect and cost, rather than for additional kidney protection. Third, some atypical eGFR trends were observed, such as increased eGFR at the second follow-up year across all drug class groups. The reason for this phenomenon should be further explored.

### Limitations

The study has its limitations. Due to the lack of albuminuria data, we could not evaluate the antihypertensive drug effectiveness on albuminuria reduction. Known confounding factors, such as dietary habits (e.g. salt intake), physical activities and other lifestyle behaviours, were not recorded in the EMR for statistical analysis.

The main reason for the lack of albuminuria/proteinuria data is the transition of the semi-quantitative urine test for proteinuria to quantitative microalbuminuria only in 2018. Furthermore, the urine tests are often unavailable due to various reasons: failure to collect urine on the spot at the lab (patients using diapers, incontinence) or inability to quantitate the urine protein/albumin due to concurrent urinary tract infection). Hence, our main health outcome focuses on more objective indicators of kidney function such as eGFR, notwithstanding that this indicator has minor confounders. Regardless of the kidney function status, the clinicians initiate or adjust the anti-hypertensive medications to optimize patients’ blood pressure control based on the local official clinical practice guidelines for hypertension management from the Ministry of Health of Singapore.

### Suggestions to real-world practice

The clinicians can leverage the results for shared clinical decision-making with their patients in selecting the appropriate first-line antihypertensive drug for those with no or early-stage CKD. Clinicians can also share with patients the effect of antihypertensive drugs on their kidney function over time to motivate and enhance treatment adherence to maintain their kidney health regardless of the drug class. The study will pave the way for further research to understand the effect of ACEIs, ARBs and OTHERS on albuminuria^36^.

## Conclusions

In conclusion, among patients with no or early-stage CKD, initiating an antihypertensive drug in ACEIs, ARBs, BBs, CCBs or Ds as monotherapy significantly delay the eGFR progression over time, regardless of drug class. However, the superiority of RASi (ACEIs/ARBs) over other antihypertensive drug classes on eGFR maintenance was not observed. This evidence may potentially inform clinical decisions on the choice of antihypertensive therapy for this patient group.

### Supplementary Information

Supplementary Information.

## Data Availability

The datasets analysed during the current study are not publicly available as they contain information that are sensitive to the study institution. They may be made available from the corresponding author on reasonable request.

## Code availability

The code used for the analyses in this study is available for reference in the GitHub repository at https://github.com/GaoQiaoPH/HTN_drug_effectiveness_on_eGFR.
