# Supplementary material for: Comparative effectiveness of first-line antihypertensive drug classes on the maintenance of estimated glomerular filtration rate (eGFR) in real world primary care
Source: Sci Rep. 2023 Dec 1;13:21225. doi: 10.1038/s41598-023-48427-4 (PMC10692108; doi:10.1038/s41598-023-48427-4)
Supplement: Supplementary file 1 — Supplementary Information. [file 41598_2023_48427_MOESM1_ESM.docx]

**Supplementary Materials**

| **Page Number** | **Contents** |
| --- | --- |
| 2 | **Table S1.** Drugs considered in each antihypertensive drug class. |
| 3 | **Table S2.** Number of various types of events related to eGFR decline in study cohort |
| 4 | **Table S3.** Baseline characteristics comparison between ACEIs and ARBs before/after weighting using IPTW |
| 6 | **Table S4.** Baseline characteristics comparison between ACEIs and OTHERS before/after weighting using IPTW |
| 8 | **Table S5.** Baseline characteristics comparison between ARBs and OTHERS before/after weighting using IPTW |
| 10 | **Table S6.** Number of individuals experienced CKD progression to a more advanced stage |
| 11 | **Figure S1.** Flow chart illustrating the derivation of the study cohorts |
| 12 | **Figure S2.** Pairwise comparison of BMI changing curve over five years |
| 13 | **Figure S3.** Comparing the SMD of baseline characteristics before/after weighting for the patients taking drugs for more than 5 years. |
| 14 | **Figure S4.** Pairwise comparison of systolic blood pressure curve over five years |
| 15 | **Figure S5.** Pairwise comparison of diastolic blood pressure curve over five years |
| 16 | **Figure S6** Hazard ratios for eGFR declining by 10% from baseline for whole cohort and subgroups |
| 17 | Details of Inverse Probability of Treatment Weighting (IPTW) |
| 18 | **Table S7** Covariates used in propensity score estimation |

**Table S 1** Drugs considered in each antihypertensive drug class.

| **Drug classes** | **Drugs** |
| --- | --- |
| Angiotensin Converting Enzyme Inhibitors (ACEIs) | Enalapril, Lisinopril, Perindopril, Captopril |
| Angiotensin II Receptor Blockers (ARBs) | Losartan, Valsartan, Telmisartan, Candesartan, Olmesartan |
| Beta-Blockers (BBs) | Atenolol, Bisoprolol |
| Calcium Channel Blockers (CCBs) | Amlodipine, Nifedipine LA |
| Diuretics (Ds) | Hydrochlorothiazide, Spironolactone, Indapamide |

**Table S 2** Number of various types of events related to eGFR decline in study cohort

| **Cohort** | **#patients** | **eGFR declines to**  **a more advanced CKD stage** | **eGFR declines by 10%** | **eGFR declines by 30%** | **eGFR declines by 50%** |
| --- | --- | --- | --- | --- | --- |
| ACEIs | 3832 (100) | 428 (11.2) | 587 (15.3) | 106 (2.8) | 12 (0.3) |
| ARBs | 3171 (100) | 429 (13.5) | 581 (18.3) | 91 (2.9) | 15 (0.5) |
| OTHERS | 12496 (100) | 941 (7.5) | 1057 (8.5) | 89 (0.7) | 9 (0.1) |
| **Total** | **19499 (100)** | **1798 (9.2)** | **2225 (11.4)** | **286 (1.5)** | **36 (0.2)** |

*The event is defined as at least two consecutive eGFR values satisfying the condition, to avoid the effect of eGFR variation. The numbers in brackets are the percentage (%) of the corresponding cohort size. The columns label "eGFR declines by x%" means that declines by x% compared to the baseline eGFR value.*

**Table S 3** Baseline characteristics comparison between ACEIs and ARBs before/after weighting using IPTW

|  | **Before Weighting** | | | **After Weighting** | | |
| --- | --- | --- | --- | --- | --- | --- |
| **Characteristics** | **ARBs** | **ACEIs** | **SMD** | **ARBs** | **ACEIs** | **SMD** |
| Age (year) | 63.4 | 63.4 | 0.00 | 63.5 | 63.5 | 0.00 |
| **Gender (%)** | | | | | | |
| Male | 46.8 | 54.7 | 0.16 | 50.4 | 50.7 | 0.01 |
| Female | 53.2 | 45.3 | -0.16 | 49.6 | 49.3 | -0.01 |
| **Race (%)** | | | | | | |
| Chinese | 79 | 76.7 | -0.05 | 78.5 | 78.8 | 0.01 |
| Malay | 9.6 | 11.5 | 0.06 | 10.2 | 9.9 | -0.01 |
| Indian | 7.1 | 8.2 | 0.04 | 7.5 | 7.5 | 0.00 |
| Others | 4.3 | 3.5 | -0.04 | 3.8 | 3.7 | -0.01 |
| **Baseline lab values** | | | | | | |
| HbA1c (%) | 7.2 | 7.2 | 0.00 | 7.2 | 7.2 | -0.01 |
| LDL-C (mmol/L) | 2.6 | 2.6 | -0.01 | 2.6 | 2.6 | -0.01 |
| Systolic BP (mmHg) | 131.1 | 128.9 | -0.14 | 130 | 129.7 | -0.02 |
| Diastolic BP (mmHg) | 71.5 | 70.7 | -0.08 | 71.1 | 71 | -0.02 |
| BMI (kg/m^2^) | 25.7 | 25.2 | -0.13 | 25.4 | 25.4 | -0.02 |
| **Comorbidities (%)** | | | | | | |
| Dyslipidemia | 90.4 | 92.9 | 0.09 | 92.1 | 92 | 0.00 |
| Diabetes | 57 | 62.9 | 0.12 | 58.9 | 59 | 0.00 |
| **Complications (%)** | | | | | | |
| Macrovascular disease | 19 | 21.8 | 0.07 | 19.6 | 20.1 | 0.01 |
| Nephropathy | 21 | 21.6 | 0.01 | 21.3 | 21.3 | 0.00 |
| Retinopathy | 18 | 18.1 | 0.00 | 17.6 | 17.7 | 0.00 |
| Foot complications | 2.1 | 2.6 | 0.03 | 2.1 | 2 | 0.00 |
| **Anti-diabetic medications (%)** | | | | | | |
| Biguanides | 50.7 | 57.4 | 0.13 | 52.9 | 53.5 | 0.01 |
| Sulfonylureas | 28.4 | 33.8 | 0.12 | 29.2 | 29.6 | 0.01 |
| DPP4 inhibitors | 3.1 | 1.4 | -0.12 | 1.0 | 1.1 | 0.01 |
| Alpha glucosidase inhibitors | 4.8 | 7 | 0.09 | 5.0 | 4.6 | -0.01 |
| Insulin | 5.7 | 6.8 | 0.04 | 5.4 | 6.0 | 0.02 |
| **Anti-hyperlipidemic medications (%)** | | | | | | |
| Hmgcoa reductase inhibitors | 87.6 | 90.1 | 0.08 | 89.2 | 89.3 | 0.00 |
| Fibric acid derivatives | 8.0 | 8.1 | 0.00 | 8.0 | 8.0 | 0.00 |
| Cholesterol absorption inhibitors | 0.2 | 0.4 | 0.04 | 0.2 | 0.3 | 0.02 |
| Bile acid sequestrants | 0.1 | 0.2 | 0.03 | 0.1 | 0.2 | 0.01 |
| **Baseline eGFR stage (%)** | | | | | | |
| Stage 1 | 51.2 | 47.1 | -0.08 | 49.1 | 49 | 0.00 |
| Stage 2 | 37.7 | 41.3 | 0.08 | 39.8 | 39.7 | 0.00 |
| Stage 3 | 10.2 | 10.9 | 0.02 | 10.5 | 10.7 | 0.01 |
| Stage 4 | 0.9 | 0.6 | -0.03 | 0.6 | 0.5 | -0.01 |
| Stage 5 | 0.1 | 0.1 | -0.02 | 0.0 | 0.1 | 0.02 |

SMD: standard mean difference

**Table S 4** Baseline characteristics comparison between ACEIs and OTHERS before/after weighting using IPTW

|  | **Before Weighting** | | | **After Weighting** | | |
| --- | --- | --- | --- | --- | --- | --- |
| **Characteristics** | **OTHERS** | **ACEIs** | **SMD** | **OTHERS** | **ACEIs** | **SMD** |
| Age (year) | 64.5 | 63.4 | -0.11 | 64 | 64.1 | 0.01 |
| **Gender (%)** | | | | | | |
| Male | 39.3 | 54.7 | 0.31 | 47.1 | 47.2 | 0.00 |
| Female | 60.7 | 45.3 | -0.31 | 52.9 | 52.8 | 0.00 |
| **Race (%)** | | | | | | |
| Chinese | 85.0 | 76.7 | -0.21 | 81.8 | 81.5 | -0.01 |
| Malay | 8.1 | 11.5 | 0.11 | 9.3 | 9.2 | 0.00 |
| Indian | 4.2 | 8.2 | 0.17 | 5.7 | 5.9 | 0.01 |
| Others | 2.7 | 3.5 | 0.05 | 3.2 | 3.4 | 0.01 |
| **Baseline lab values** | | | | | | |
| HbA1c (%) | 7.1 | 7.2 | 0.10 | 7.1 | 7.1 | 0.00 |
| LDL-C (mmol/L) | 2.8 | 2.6 | -0.32 | 2.7 | 2.7 | -0.01 |
| Systolic BP (mmHg) | 132.4 | 128.9 | -0.22 | 130.9 | 130.5 | -0.03 |
| Diastolic BP (mmHg) | 71.9 | 70.7 | -0.12 | 71.6 | 71.3 | -0.02 |
| BMI (kg/m^2^) | 25.3 | 25.2 | -0.02 | 25.3 | 25.3 | 0.00 |
| **Comorbidities (%)** | | | | | | |
| Dyslipidemia | 83.9 | 92.9 | 0.28 | 90.1 | 89.9 | -0.01 |
| Diabetes | 14.3 | 62.9 | 1.15 | 34.3 | 35.2 | 0.02 |
| **Complications (%)** | | | | | | |
| Macrovascular disease | 17.2 | 21.8 | 0.12 | 21.4 | 20.9 | -0.01 |
| Nephropathy | 11.0 | 21.6 | 0.29 | 13.9 | 14.5 | 0.02 |
| Retinopathy | 5.1 | 18.1 | 0.41 | 9.8 | 10.6 | 0.02 |
| Foot complications | 0.9 | 2.6 | 0.13 | 1.5 | 1.3 | -0.01 |
| **Anti-diabetic medications (%)** | | | | | | |
| Biguanides | 12.8 | 57.4 | 1.06 | 30.8 | 31.6 | 0.02 |
| Sulfonylureas | 6.5 | 33.8 | 0.72 | 15.6 | 16.5 | 0.02 |
| DPP4 inhibitors | 0.4 | 1.4 | 0.11 | 0.7 | 0.8 | 0.01 |
| Alpha glucosidase inhibitors | 1.0 | 7.0 | 0.31 | 2.0 | 2.3 | 0.02 |
| Insulin | 0.8 | 6.8 | 0.32 | 1.4 | 1.7 | 0.02 |
| **Anti-hyperlipidemic medications (%)** | | | | | | |
| Hmgcoa reductase inhibitors | 80.3 | 90.1 | 0.28 | 87 | 86.9 | 0.00 |
| Fibric acid derivatives | 6.2 | 8.1 | 0.07 | 7.4 | 7.0 | -0.02 |
| Cholesterol absorption inhibitors | 0.2 | 0.4 | 0.05 | 0.2 | 0.3 | 0.03 |
| Bile acid sequestrants | 0.1 | 0.2 | 0.03 | 0.1 | 0.1 | 0.01 |
| **Baseline eGFR stage (%)** | | | | | | |
| Stage 1 | 49.6 | 47.1 | -0.05 | 47.8 | 48.2 | 0.01 |
| Stage 2 | 44.4 | 41.3 | -0.06 | 44.7 | 43.7 | -0.02 |
| Stage 3 | 5.8 | 10.9 | 0.18 | 7.1 | 7.6 | 0.02 |
| Stage 4 | 0.2 | 0.6 | 0.07 | 0.3 | 0.4 | 0.01 |
| Stage 5 | 0.0 | 0.1 | 0.01 | 0.0 | 0.1 | 0.02 |

SMD: standard mean difference

**Table S 5** Baseline characteristics comparison between ARBs and OTHERS before/after weighting using IPTW

|  | **Before Weighting** | | | **After Weighting** | | |
| --- | --- | --- | --- | --- | --- | --- |
| **Characteristics** | **OTHERS** | **ARBs** | **SMD** | **OTHERS** | **ARBs** | **SMD** |
| Age (year) | 64.5 | 63.4 | -0.10 | 63.9 | 63.8 | -0.01 |
| **Gender (%)** | | | | | | |
| Male | 39.3 | 46.8 | 0.15 | 43.3 | 43.4 | 0.00 |
| Female | 60.7 | 53.2 | -0.15 | 56.7 | 56.6 | 0.00 |
| **Race (%)** | | | | | | |
| Chinese | 85 | 79 | -0.16 | 82.9 | 82.8 | 0.00 |
| Malay | 8.1 | 9.6 | 0.06 | 8.5 | 8.3 | -0.01 |
| Indian | 4.2 | 7.1 | 0.13 | 5.3 | 5.7 | 0.02 |
| Others | 2.7 | 4.3 | 0.09 | 3.2 | 3.2 | 0.00 |
| **Baseline lab values** | | | | | | |
| HbA1c (%) | 7.1 | 7.2 | 0.09 | 7.1 | 7.1 | 0.00 |
| LDL-C (mmol/L) | 2.8 | 2.6 | -0.30 | 2.7 | 2.7 | -0.02 |
| Systolic BP (mmHg) | 132.4 | 131.1 | -0.08 | 131.8 | 131.4 | -0.03 |
| Diastolic BP (mmHg) | 71.9 | 71.5 | -0.04 | 71.9 | 71.7 | -0.02 |
| BMI (kg/m^2^) | 25.3 | 25.7 | 0.11 | 25.6 | 25.6 | 0.00 |
| **Comorbidities (%)** | | | | | | |
| Dyslipidemia | 83.9 | 90.4 | 0.19 | 88.2 | 88.2 | 0.00 |
| Diabetes | 14.3 | 57.0 | 1.00 | 31.4 | 32.6 | 0.03 |
| **Complications (%)** | | | | | | |
| Macrovascular disease | 17.2 | 19.0 | 0.05 | 18.9 | 18.7 | -0.01 |
| Nephropathy | 11.0 | 21.0 | 0.28 | 12.9 | 13.8 | 0.03 |
| Retinopathy | 5.1 | 18.0 | 0.41 | 9.1 | 9.9 | 0.03 |
| Foot complications | 0.9 | 2.1 | 0.10 | 1.3 | 1.4 | 0.01 |
| **Anti-diabetic medications (%)** | | | | | | |
| Biguanides | 12.8 | 50.7 | 0.89 | 28.3 | 29 | 0.02 |
| Sulfonylureas | 6.5 | 28.4 | 0.60 | 14.3 | 14.7 | 0.01 |
| DPP4 inhibitors | 0.4 | 3.1 | 0.21 | 0.3 | 0.3 | 0.00 |
| Alpha glucosidase inhibitors | 1.0 | 4.8 | 0.23 | 1.9 | 2.2 | 0.02 |
| Insulin | 0.8 | 5.7 | 0.28 | 0.7 | 1.4 | 0.07 |
| **Anti-hyperlipidemic medications (%)** | | | | | | |
| Hmgcoa reductase inhibitors | 80.3 | 87.6 | 0.2 | 84.6 | 84.7 | 0.00 |
| Fibric acid derivatives | 6.2 | 8.0 | 0.07 | 6.9 | 7.1 | 0.00 |
| Cholesterol absorption inhibitors | 0.2 | 0.2 | 0.01 | 0.1 | 0.2 | 0.02 |
| Bile acid sequestrants | 0.1 | 0.1 | 0.00 | 0.1 | 0.1 | 0.00 |
| **Baseline eGFR stage (%)** | | | | | | |
| Stage 1 | 49.6 | 51.2 | 0.03 | 51 | 50.9 | 0.00 |
| Stage 2 | 44.4 | 37.7 | -0.14 | 42.1 | 41.4 | -0.01 |
| Stage 3 | 5.8 | 10.2 | 0.16 | 6.7 | 7.3 | 0.02 |
| Stage 4 | 0.2 | 0.9 | 0.09 | 0.2 | 0.3 | 0.02 |
| Stage 5 | 0.0 | 0.1 | 0.04 | 0.0 | 0.1 | 0.03 |

SMD: standard mean difference

**Table S6** Number of individuals experienced CKD progression to a more advanced stage

(a) ACEIs

| **To stage**  **From stage** | **G1** | **G2** | **G3** | **G4** | **G5** | **(Total)** |
| --- | --- | --- | --- | --- | --- | --- |
| **G1** | 1321 (73.2) | 459 (25.4) | 23 (1.3) | 2 (0.1) | / | 1805 (100.0) |
| **G2** | / | 1258 (79.4) | 324 (20.5) | 2 (0.1) | / | 1584 (100.0) |
| **G3** | / | / | 362 (86.6) | 55 (13.2) | 1 (0.2) | 418 (100.0) |
| **G4** | / | / | / | 22 (95.7) | 1 (4.3) | 23 (100.0) |
| **G5** | / | / | / | / | 2 (100.0) | 2 (100.0) |

(b) ARBs

| **To stage**  **From stage** | **G1** | **G2** | **G3** | **G4** | **G5** | **(Total)** |
| --- | --- | --- | --- | --- | --- | --- |
| **G1** | 1152 (71.0) | 455 (28.1) | 15 (0.9) | / | / | 1622 (100.0) |
| **G2** | / | 922 (77.2) | 266 (22.3) | 6 (0.5) | / | 1194 (100.0) |
| **G3** | / | / | 278 (86.1) | 45 (13.9) | / | 323 (100.0) |
| **G4** | / | / | / | 24 (85.7) | 4 (14.3) | 28 (100.0) |
| **G5** | / | / | / | / | 4 (100.0) | 4 (100.0) |

(c) OTHERS

| **To stage**  **From stage** | **G1** | **G2** | **G3** | **G4** | **G5** | **(Total)** |
| --- | --- | --- | --- | --- | --- | --- |
| **G1** | 4745 (76.5) | 1418 (22.9) | 37 (0.6) | / | / | 6200 (100.0) |
| **G2** | / | 4958 (89.4) | 579 (10.4) | 6 (0.1) | / | 5543 (100.0) |
| **G3** | / | / | 667 (91.9) | 56 (7.7) | 3 (0.4) | 726 (100.0) |
| **G4** | / | / | / | 19 (79.2) | 5 (20.8) | 24 (100.0) |
| **G5** | / | / | / | / | 3 (100.0) | 3 (100.0) |

The numbers in each cell are n (%). The cell with same *from stage* and *to stage* includes the counts of individuals remain in the same CKD stage during the observation period.

**
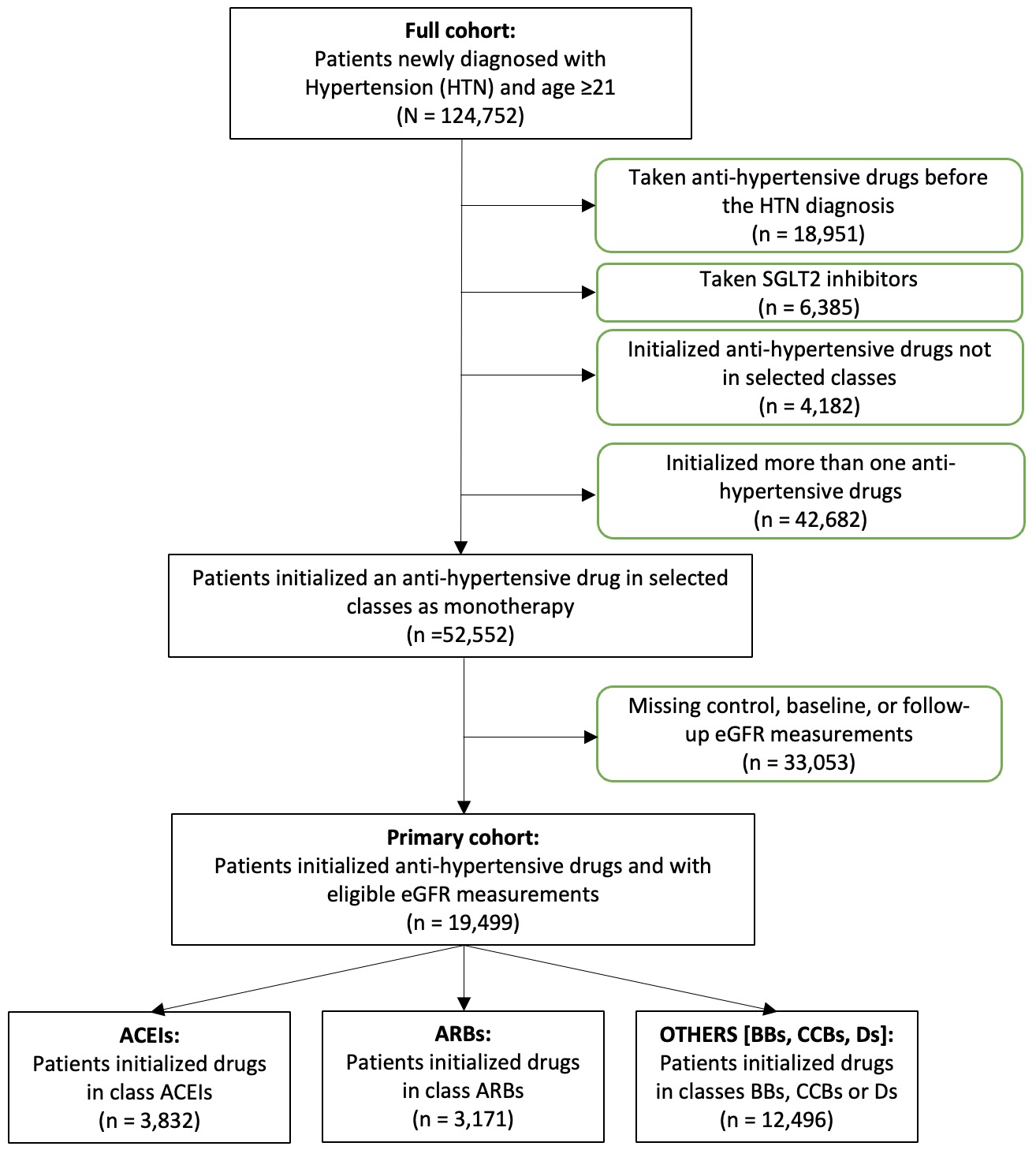
**

**Figure S1** Flow chart illustrating the derivation of the study cohorts


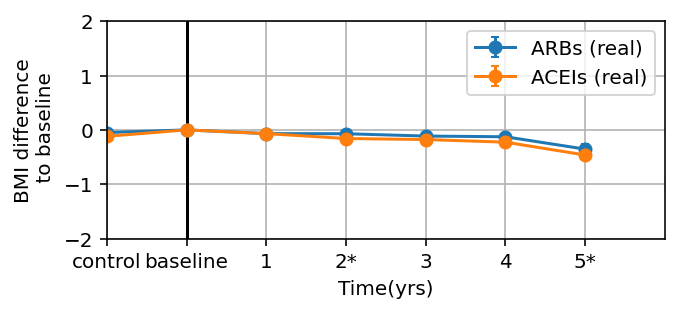

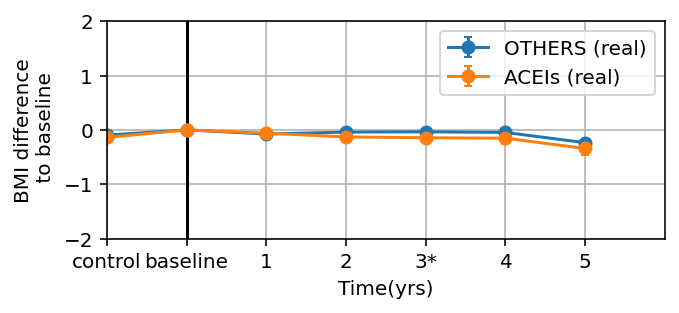


1. ACEIs vs. ARBs (b) ACEIs vs. OTHERS


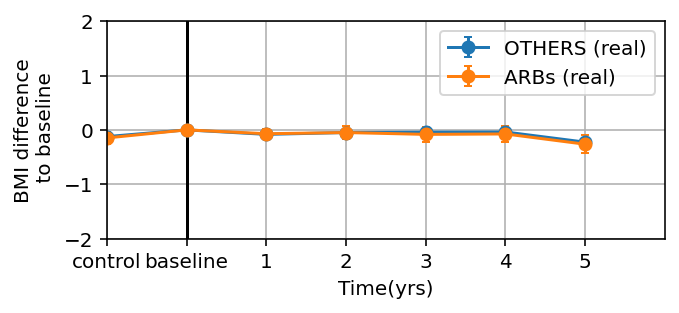


(c) ARBs vs. OTHERS

**Figure S2** Pairwise comparison of BMI curve over five years

The asterisks besides the time indicate that the corresponding means are statistically significant different (P<0.05). *The error bar of each data point depicts the 95% confidence interval.*

**
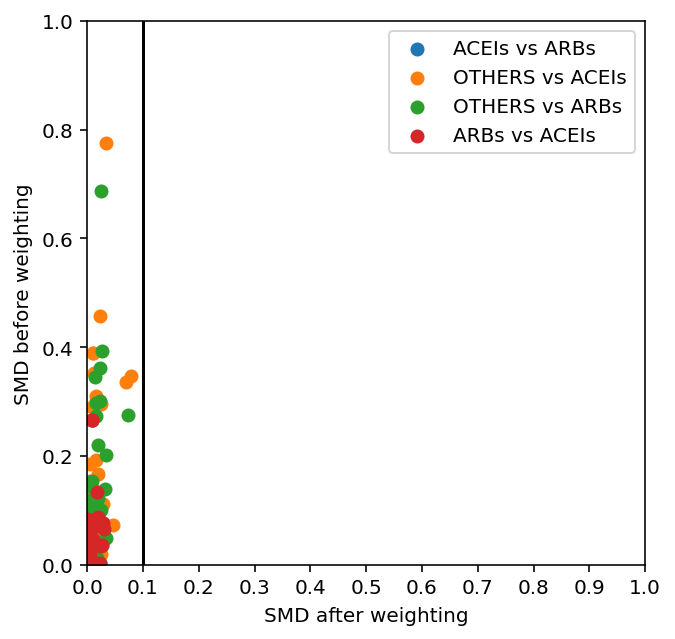
**

**Figure S3** SMD before/after the weighting for the patients taking drugs for more than 5 years


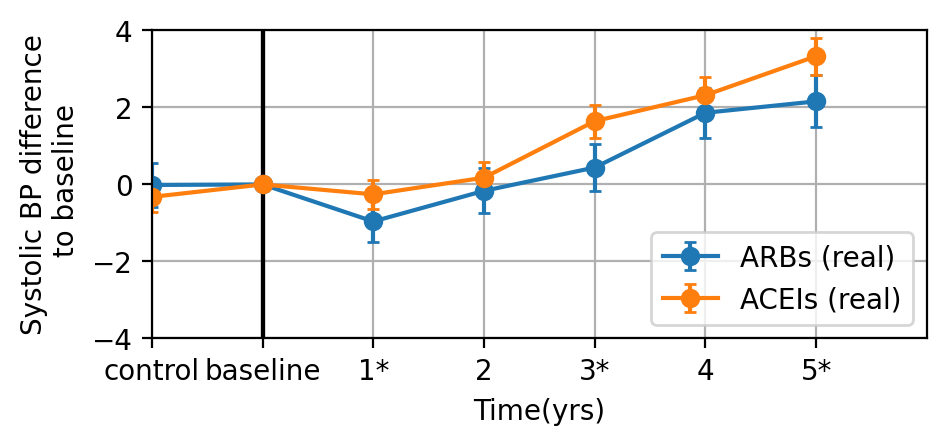

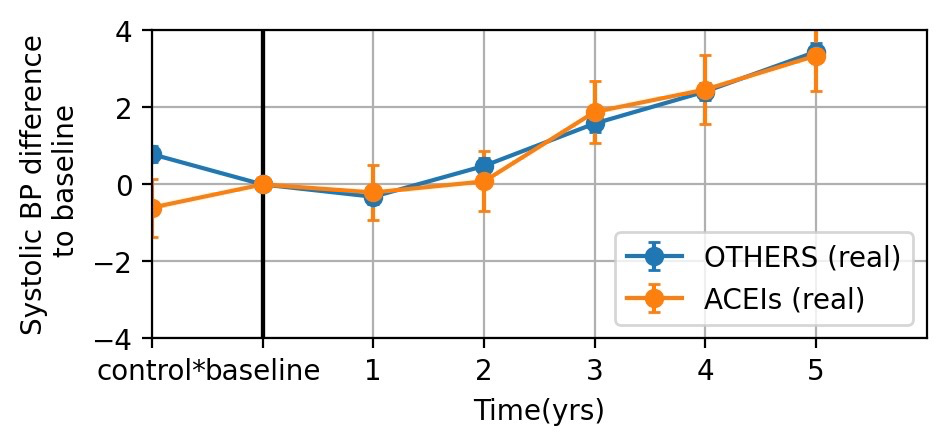


1. ACEIs vs. ARBs (b) ACEIs vs. OTHERS
2.
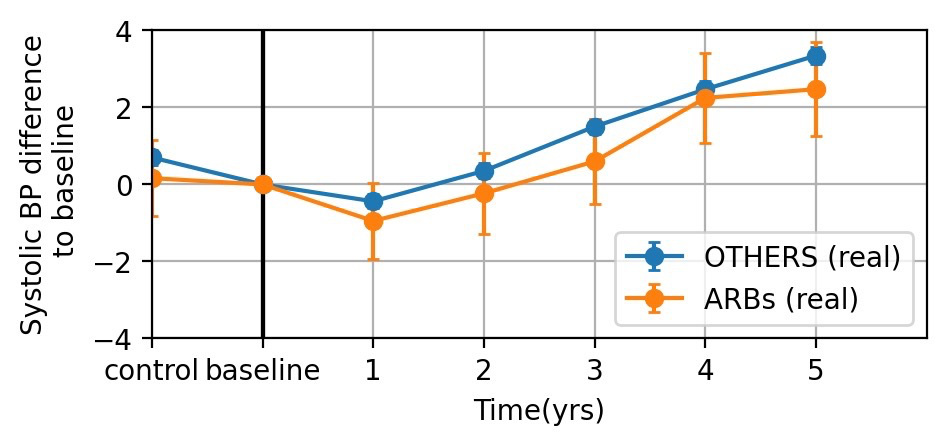


(c) ARBs vs. OTHERS

**Figure S4** Pairwise comparison of systolic blood pressure curve over five years

The asterisks besides the time indicate that the corresponding means are statistically significant different (P<0.05). *The error bar of each data point depicts the 95% confidence interval.*


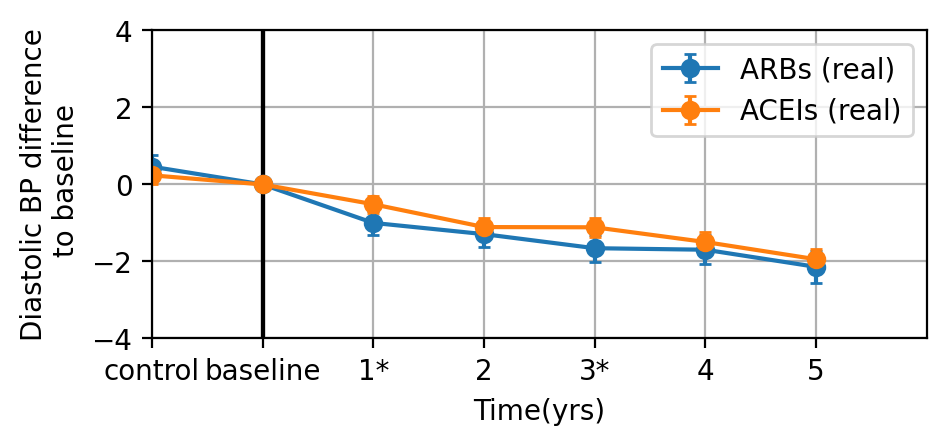

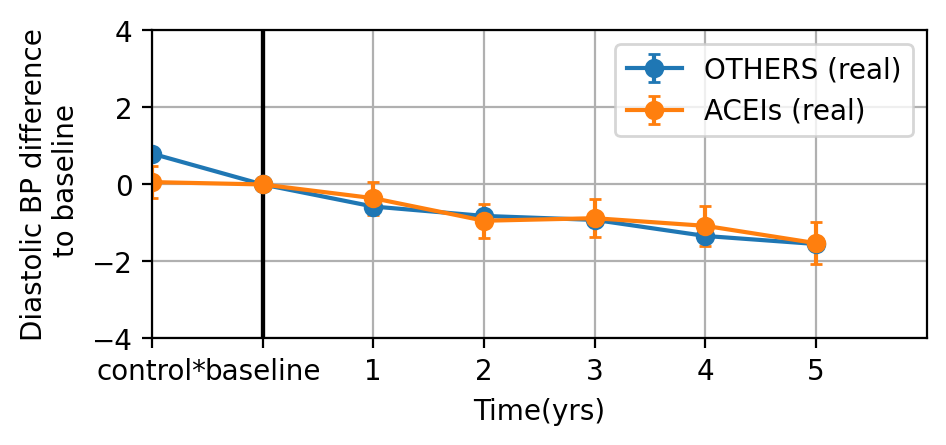


1. ACEIs vs. ARBs (b) ACEIs vs. OTHERS


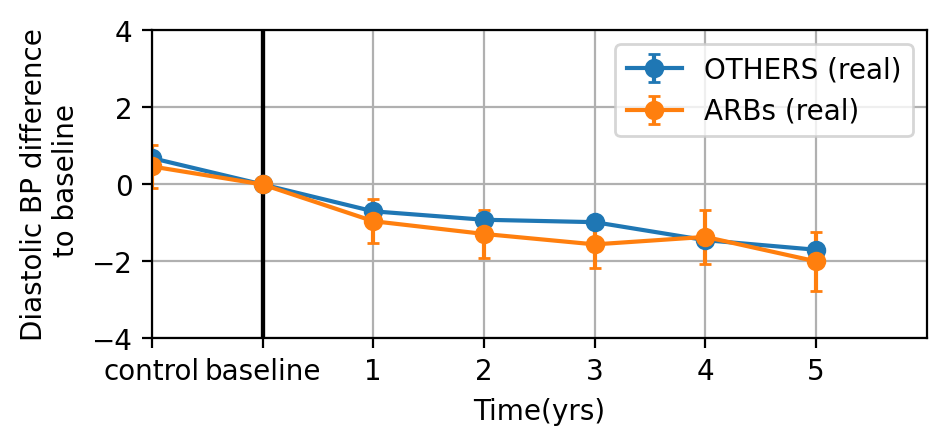


(c) ARBs vs. OTHERS

**Figure S5** Pairwise comparison of diastolic blood pressure curve over five years

The asterisks besides the time indicate that the corresponding means are statistically significant different (P<0.05). *The error bar of each data point depicts the 95% confidence interval.*


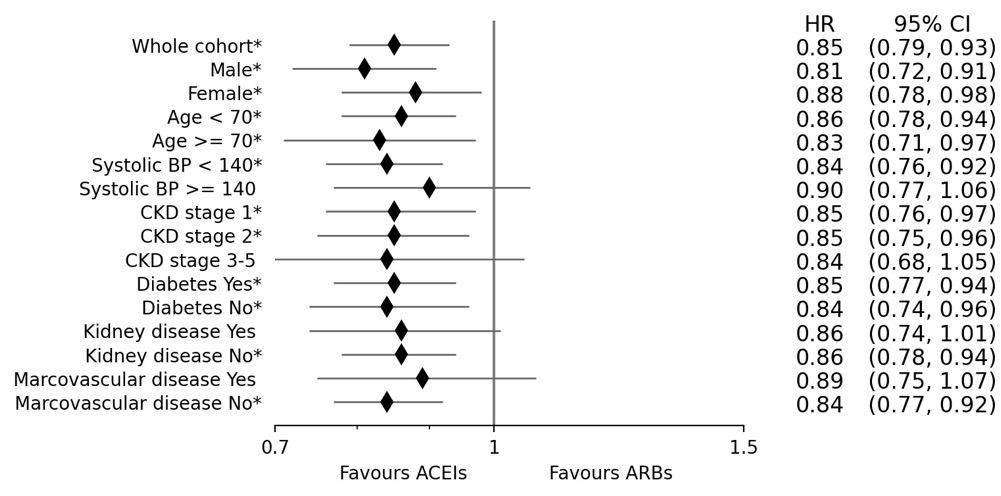


(a). ACEIs vs. ARBs


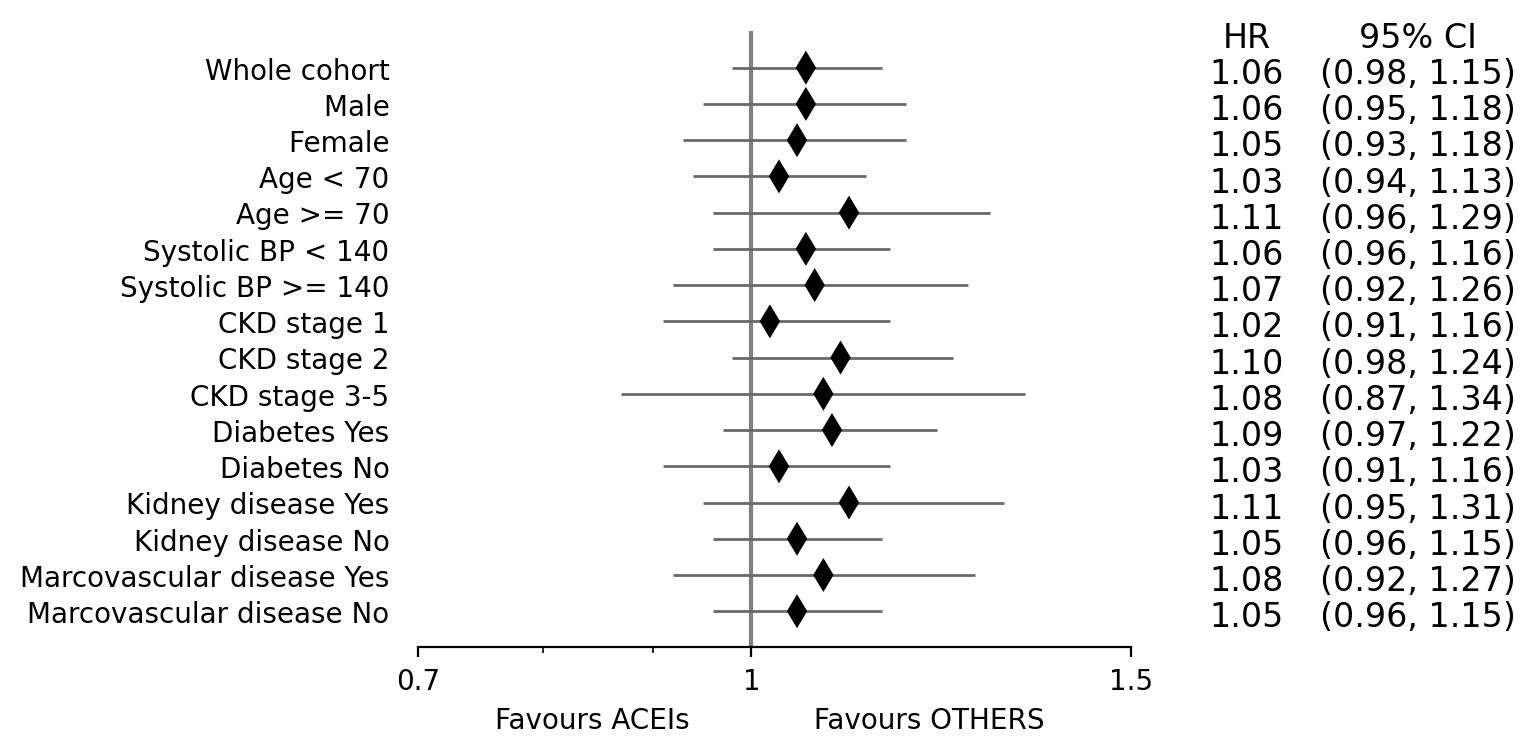


(b). ACEIS vs. OTHERS


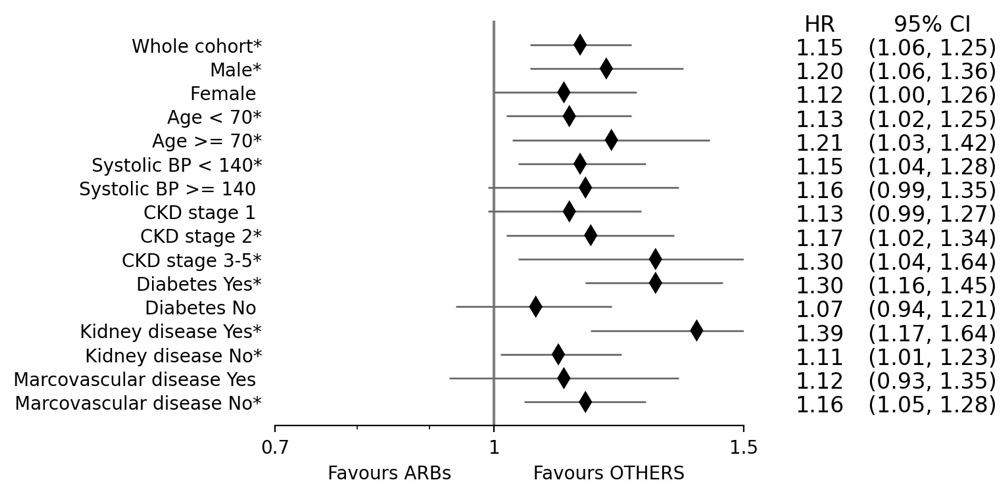


(c). ARBs vs OTHERS

**Figure S6** Hazard ratios for eGFR declining by 10% from baseline for whole cohort and subgroups divided according to sex, age, systolic blood pressure, CKD stage, diabetes, kidney disease and macrovascular disease.

*The asterisks besides the labels indicate that the corresponding HR are statistically significant different (P<0.05).*

**Details of Inverse Probability of Treatment Weighting (IPTW)**

IPTW stands as a robust methodology within causal inference, aiming to neutralize confounding variables at baseline during the evaluation of treatment effectiveness across two distinct treatment cohorts.

The key steps of this methodology encompass:

- **Estimation of Propensity Scores:** For individuals receiving disparate treatments, we harness baseline covariates to compute the likelihood of each participant receiving their respective treatment, given their covariates. This calculated probability is referred to as the propensity score (PS). We used a multivariate logistic regression model for PS estimation, incorporating L2 regularization and class weighting. The L2 regularization, set with a strength of $\lambda$ equal to 2, helps to prevent overfitting. The class weights were inversely proportional to the frequencies of the classes in our data, addressing potential model bias towards more common classes. This approach is particularly important in cases of class imbalance. For detailed information on the covariates included in the PS estimation, please refer to Table S7.
- **Verification of the Positivity Assumption:** Utilizing the estimated propensity scores, we employ a histogram to scrutinize the degree of overlap between the propensity scores of the two treatment groups. This enables us to pinpoint individuals exhibiting insufficient overlap in their propensity scores, signaling a potential breach of the positivity assumption. This assumption mandates that every participant, irrespective of their covariate profile, must possess a non-zero probability of being assigned to either treatment category. In essence, the absence of treatment assignment possibilities for certain individuals implies an inherent inability to adjust for confounders, as we lack corresponding outcome data from the alternate treatment group for these specific cases. In IPTW, the extent of propensity score overlap serves as a criterion to identify individuals infringing upon the positivity assumption. For this study, individuals residing outside the 2nd to 98th percentile range of the overlapped propensity score distribution were excluded from subsequent analyses.
- **Application of Inverse Probability of Treatment Weighting**: Subsequent to the exclusion process, the remaining individuals were assigned weights based on the inverse of their propensity scores corresponding to the received treatment. These weights were adjusted by marginal probability of treatment aiming to stabilizing the weights and enhancing the reliability of the causal effect estimation.

**Table S7** Covariates used in propensity score estimation

| **Category** | **Covariates** |
| --- | --- |
| Demographics | Age, Gender, Race |
| Baseline lab values | HbA1c, LDL-C, Systolic BP, Diastolic BP, BMI |
| Kidney function | Baseline CKD stage |
| Comorbidities | Dyslipidaemia, Diabetes |
| Complications | Macrovascular disease, Nephropathy, Retinopathy, Foot complications |
| Anti-diabetic medications | Biguanides, Sulfonylureas, DPP4 inhibitors, Alpha glucosidase inhibitors, Insulin |
| Anti-hyperlipidemic medications | HMG-CoA reductase inhibitors, Fibric acid derivatives, Cholesterol absorption inhibitors, Bile acid sequestrants |
